# Observation of MRI‐Based Endolymphatic Hydrops and Clinical Symptoms in Patients With Definite Meniere′s Disease Over Time: A Case Series

**DOI:** 10.1155/bmri/9060023

**Published:** 2026-09-03

**Authors:** Christoph J. Pfeiffer, Hans-Björn Gehl, Alexander Kilgue, Conrad Riemann, Lars-Uwe Scholtz, Dina Voeltz, Ingo Todt

**Affiliations:** ^1^ Department of Otolaryngology, Head and Neck Surgery, Klinikum Bielefeld Mitte, Medical School OWL, Bielefeld University, Bielefeld, Germany, uni-bielefeld.de; ^2^ Department of Otolaryngology and Facial Plastic Surgery, Alexianer St. Gertrauden Hospital, Berlin, Germany; ^3^ Brandenburg Medical School Theodor Fontane (MHB), Neuruppin, Germany; ^4^ Department of Otolaryngology, Hospital Luis Vernaza de la Junta de Beneficencia, Guayaquil, Ecuador; ^5^ Bielefeld University, Biostatistics and Medical Biometry, Medical School OWL, Bielefeld, Germany, uni-bielefeld.de

**Keywords:** endolymphatic hydrops, hydrops sequence, Ménière′s disease, MRI, vertigo

## Abstract

**Purpose:**

The assessment of endolymphatic hydrops (ELH) with specific MRI sequences underlines the ability to evaluate intralabyrinthine changes objectively and radiologically. The visualization of ELH by MRI allows correlations to be made with clinical patterns and supports the diagnosis of Ménière′s disease (MD). Because MD cannot be assumed to be a static clinical condition, follow‐up observations are of particular clinical interest. Our examination aimed to compare the grading of MRI‐based ELH in patients over time, considering clinical symptoms and possible interventions.

**Material and Methods:**

Two MRI hydrops examinations and their gradings, from 11 patients with clinically definite MD, were compared in each case. The clinical pattern before and after conservative therapy or endolymphatic sac surgery was assessed over time using linear mixed regression.

**Results:**

In 9 out of 11 patients, we observed an aligned trend in the MRI‐based grading and hearing loss during an attack of MD. There was an increase in both parameters in eight patients. In one patient, an unchanged grading was observed. In one case, decrease was noted. In three cases there was a change of ELH, although the average hearing loss did not change.

**Conclusion:**

The MRI‐based ELH may change during MD. Influencing variables and the correlation with clinical symptoms might be of great clinical interest regarding the success of therapy and the subjective complaints of patients. The different treatment options contribute to varying degrees to slowing the progression of MD.

## 1. Introduction

The first description of endolymphatic hydrops (ELH) was demonstrated postmortem in temporal bone specimens in 1938 [1, 2].

It describes an increase of the endolymphatic rooms in the inner ear [1].

Because specific MRI sequences have become available, it has been possible to visualize ELH not only postmortem but also in vivo [3]. In a further development of this examination technique, it became possible in everyday clinical evaluation to use imaging for the objective assessment of the inner ear and its pathologies in living patients. In this context, ELH is a pathology that can be related to the clinical symptoms like aural fullness, hearing loss, tinnitus and vertigo [4].

On the basis of the otological symptoms, hydrops is distinguished from other otological diseases when it occurs. Due to the varying otological symptoms, ELH is associated with other (neuro‐)otological diseases. These diseases include Ménière′s disease (MD) and large vestibular aqueduct syndrome (LVAS); additionally, exposure to noise trauma is also discussed [5–7].

Although there are many hypotheses, the definitive etiology of MD is unidentified [8]. MD has a wide range of prevalence between different regions in the world [9–11].

In patients with MD, the ear in which the clinical symptoms can be attributed is called the index ear [12]. The MRI‐based visualization of an ELH helps to support the diagnosis of MD [13, 14]. Both MD and ELH can occur bilaterally in 10%–50% [15, 16].

Related to its fluctuating clinical variation it cannot be expected to present a constant clinical MRI pattern. Therefore, subsequent monitoring is of great clinical importance. Various research groups have proposed different grades of ELH. They distinguished none, mild, and significant ELH [17]. There also have been distinctions both in terms of scoring and, in some cases, a distinction between the vestibular and cochlear portions or the expression of the endolymphatic duct [13, 14, 18–21].

Different studies showed a correlation between the ELH in MRI pattern and the MD‐related clinical appearance. Even a dependence the ELH on the chosen therapy and partly also on the follow‐up care is discussed [18, 21–23]. There are also suggestions that hearing loss can be predicted by increasing ELH grades, whereas other authors cannot comprehend hearing loss caused by ELH [24, 25]. In many cases, MD leads to functional deafness in the affected ear, and cochlear implantation is the treatment of choice to restore hearing. If MD occurs on both sides, ELH on the nonimplanted side can also be monitored with MRI although there are artifacts [26].

A correlation between ELH grades and the severity of clinical symptoms was discussed [27].

So far, it is thought that ELH, like MD, gets worse over time until the disease burns itself out. In this context, hearing loss leads to functional deafness. The behavior of ELH over a longer period of time has been little studied [21].

Various therapeutic approaches to MD can lead to changes in the ELH [28, 29].

The present study is aimed at a longitudinal observation of our patients. For this purpose, we used an MRI‐based grading system of the ELH. In addition to the imaging findings, we also collected clinical data and monitored these findings over time, just as we did with the imaging findings. Furthermore, we correlated the findings with one another.

## 2. Material and Methods

### 2.1. Patients

Retrospectively, 11 patients (7 males and 4 females) with clinically definite MD who presented twice to our department between 2015 and 2024 were analyzed. The patients′ diagnoses were made based on the Barany Society′s criteria for definitive MD [30, 31]. Patients with probable MD or other vestibular disorders were excluded. Another exclusion criterion was patients with incomplete imaging.

The mean age of the patients was 52.1 years, and the median age was 52.0 years. All patients underwent a micro‐otoscopy, a pure tone audiometry (PTA), a temporal bone CT, a nystagmography, caloric testing, and an MRI with hydrops sequence at both appointments. A temporal bone CT scan was only performed at the first appointment. The results of these examinations excluded a differential diagnosis for the symptoms and determined the index ear of MD.

Before the first hospitalization in our clinic, the patients′ primary treatment consisted of conservative therapy with a low salt diet and/or betahistine medication, a treatment with multiple doses of intravenous corticosteroids, or endolymphatic sac surgery [32].

The standard treatment plan for intravenous corticosteroids was based on methylprednisolone. A daily dose of 250 mg was administered for 2 days. This was followed by 3 days with a daily dose of 150 mg. Methylprednisolone was administered for a further 5 days, with the dose starting at 100 mg and being reduced by 20 mg per day. In total, methylprednisolone was administered for 10 days.

In one patient, a surgical procedure was performed several times in addition to the conservative therapy. The patient underwent repeated endolymphatic sac drainage surgery before another hospitalization and the first MRI.

During this first hospitalization, the patient underwent therapy with intravenous corticosteroids in a descending dose regimen. Additionally, an MRI was performed. During the time between the MRI scans, all patients received additional conservative therapy with betahistine. There was no operative intervention between the MRI scans in any patient.

A Ménière′s attack under the continuation of conservative therapy, as well as a subsequent hospitalization in our clinic, was required to include the patients in this study. An MRI scan was performed during the second hospitalization. The average time between examinations was 866 days.

In all patients, an index ear was defined as the ear with symptoms of hearing loss, tinnitus, and first onset of symptoms [12]. There was no exclusion for patients with both‐sided MD. In bilateral cases the index ear was defined as the ear in which the beginning of symptoms was earlier.

The study was conducted in accordance with the Declaration of Helsinki. The local ethics committee approved this study (Ärztekammer Westfalen‐Lippe, University of Münster Faculty of Medicine, Bielefeld University, Medical Faculty—Reference: 2022‐314‐f‐S). All patients agreed to participate in the study and gave their written informed consent.

### 2.2. Definition of ELH Index

ELH severity in both MRI scans was recorded according to the grading for vestibular hydrops of Bernaerts et al. and Gürkov et al. for cochlea hydrops. In these gradings, a distinction between vestibular and cochlear hydrops with a value between 0 and 3 can be made [14, 18, 19]. Bernaerts et al. presented a four‐stage grading system for vestibular hydrops. In Stage 0, a normally sized utricle and saccule are visible. Stage 1 shows an enlarged saccule which is at least the same size as the utricle. In Stage 2, the saccule and utricle appear connected and cannot be distinguished. The perilymphatic space is still recognizable as a small surrounding. In Stage 3, the perilymphatic space is obliterated completely [18].

Gürkov et al. described the grading for cochlea hydrops. A normal cochlea without enlarged endolymphatic spaces is shown in Grade 0. In Grade 1, there is an enlarged endolymphatic space. In addition to the enlarged endolymphatic space, changes in the perilymphatic space can be observed in Grades 2 and 3. Although the perilymphatic space is still visible in a semicircular form in Grade 2, it appears leveled in Grade 3 [14].

Based on the grading for the index ear and recording, an index between 0 and 6 can be calculated. The index was used to follow‐up.

vestibular+cochlear=ELH index 06−

In cases of bilateral appearance, the index was calculated for each ear.

### 2.3. MRI Imaging

All scans were performed in a 3T‐MRI system (Ingenia, Philips, Best, The Netherlands) with a 16‐channel array head coil. The used contrast agent was Gadobutrol, given intravenously at a dose of 0.2 mL/kg 4 h before scanning. To detect a hydrops an inversion recovery sequence was used. The standard parameters were resolution 384 × 384, TR 6000, TE 177, and TI 2000.

A three‐dimensional fluid‐attenuated inversion recovery (3D‐FLAIR) sequence was included in the imaging protocol. Thus, other pathologies such as infarction, vestibular schwannomas, or inner ear malformations could also be discarded.

### 2.4. Air Conducted Hearing Loss

A pure‐tone audiogram (PTA) was performed at the first hospitalization. At the time of the following attack, a second PTA was done. The average hearing loss of the frequencies of 250, 500, and 1000 Hz was determined in both. A change in the average hearing loss was defined by ≥ 10 dB HL.

### 2.5. Statistical Analysis

All analysis was performed in R (RStudio Team) based on Excel spreadsheets (Microsoft, Redmond, United States). A comparison between the index of both time points and the hearing loss was made for each patient using a linear mixed regression model adjusted for the covariates age and sex.

## 3. Results

All patients underwent different treatments and therapy schemes before the first hospital stay in our department. In consequence of these various treatments, ELH index, the average hearing loss for the index ear and the reaction in caloric testing were the three main items taken into comparison. Therefore, these items at the time of the first stay in our clinic define a baseline for the statistical analysis the clinical findings at the time of the first hospitalization were used. The baseline values serve as the basis for the comparison and are intended to help standardize the changes observed over time.

On basis of the first MRI scan and the symptoms of the patients an index ear was defined in all patients. In seven patients, the index ear was right‐sided, whereas in four patients, it was the left ear. There was no bilaterality among the patients. The patients′ first symptoms of MD began 1.5–6 years before the first hospitalization in our department. The basic data for our study population is shown in Table 1. More data is available in the supporting information (Available here).

**Table 1:** Basic data of study population.

Number of patients	11
Male/female	7/4
Age (year)	52.1 (35.4–65.8)
Duration of illness (year)	3.0 (1.5–6)
Days between examinations	856
Therapy	
Corticosteroid infusion	11
Betahistine	11
Saccotomy	1

**Figure 1:**
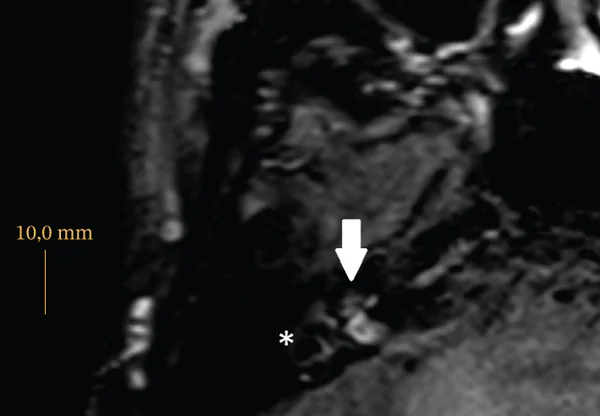
Axial section of the 3D FLAIR sequence of an MRI at the first time point without ELH in the vestibulum (white star) or in the cochlea (white arrow) with a scale bar.

**Figure 2:**
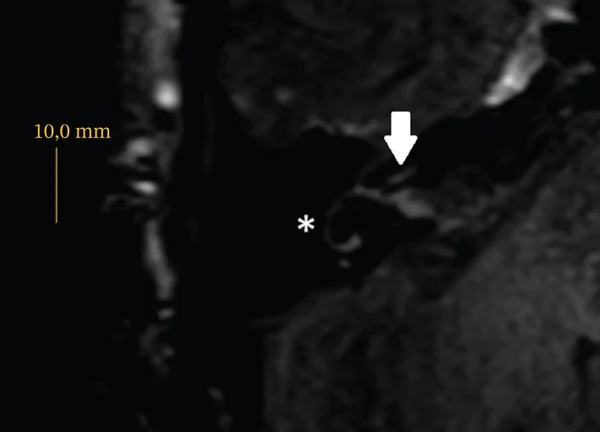
Axial slice of the 3D FLAIR sequence of the MRI of the same patient as in Figure 1 at the second time point with ELH Grade 2 in the vestibule (white star) and Grade 2 in the cochlea (white arrow) with a scale bar.

Between the two consecutive MRI scans only conservative therapy was applied. The first MRI scan showed no ELH in two patients. The grading progressed in nine patients, whereas one patient showed decrease, and one patient showed an unchanged pattern of the MRI‐based grading of the ELH. An example for the measurement is given in Figures 1 and 2. The exact results of both MRI examinations and the calculated ELH indices for each patient are given in Table 2.

**Table 2:** MRT‐evaluated endolymphatic hydrops grading, calculation, and change of index for all patients and both performed MRI scans.

Patient no.	Index ear	First MRI examination					Second MRI examination					Index‐change
		Right		Left		Index 1	Right		Left		Index 2	
		Vestibular	Cochlear	Vestibular	Cochlear		Vestibular	Cochlear	Vestibular	Cochlear		
1	Right	0	0	0	0	0	1	2	0	0	3	3
2	Right	1	0	0	0	1	2	2	0	0	4	3
3	Right	0	0	0	0	0	2	2	0	1	4	4
4	Left	0	0	2	1	3	0	0	2	2	4	1
5	Left	0	0	2	1	3	0	0	2	2	4	1
6	Right	1	0	0	0	1	1	2	0	2	3	2
7	Left	0	0	0	1	1	0	0	0	1	1	0
8	Right	0	1	0	0	1	3	2	0	0	5	4
9	Left	0	0	2	2	4	0	0	3	3	6	2
10	Right	1	0	1	0	1	0	0	1	0	0	‐1
11	Right	1	1	0	0	2	3	2	0	0	5	3

*Note:* Endolymphatic hydrops index of the index ear = vestibular + cochlear (0–6).

Patient no. 4 underwent endolymphatic sac surgery before the first MRI scan.

In 9 out of 11 patients, there is an analogy in the course of the ELH index to the development of the average hearing loss in the three frequencies investigated in the index ear. The PTA threshold during the first and the second examination is shown in Table 3.

**Table 3:** Pure‐tone audiogram (PTA) threshold in dB HL in first and second audiogram for all patients.

Patient no	Index ear		Right ear					Left ear				
			0.25 kHz	0.5 kHz	1 kHz	2 kHz	4 kHz	0.25 kHz	0.5 kHz	1 kHz	2 kHz	4 kHz
1	Right	Audiogram 1	**60**	**55**	**60**	**40**	**35**	10	5	10	10	20
		Audiogram 2	**80**	**75**	**70**	**55**	**65**	10	10	15	10	30
2	Right	Audiogram 1	**55**	**55**	**50**	**30**	**35**	40	50	45	35	35
		Audiogram 2	**85**	**75**	**70**	**65**	**75**	45	50	50	55	35
3	Right	Audiogram 1	**20**	**15**	**15**	**20**	**40**	20	15	15	20	40
		Audiogram 2	**50**	**45**	**35**	**45**	**55**	20	20	20	30	45
4	Left	Audiogram 1	15	10	15	10	10	**50**	**50**	**55**	**45**	**55**
		Audiogram 2	25	15	15	15	20	**60**	**60**	**65**	**65**	**65**
5	Left	Audiogram 1	15	15	15	20	25	**40**	**30**	**30**	**35**	**35**
		Audiogram 2	15	15	15	20	30	**55**	**50**	**40**	**40**	**85**
6	Right	Audiogram 1	**40**	**30**	**25**	**25**	**30**	15	15	15	20	25
		Audiogram 2	**50**	**50**	**50**	**45**	**50**	10	15	15	15	15
7	Left	Audiogram 1	10	20	25	15	10	**70**	**70**	**70**	**50**	**40**
		Audiogram 2	40	40	30	20	15	**75**	**75**	**70**	**60**	**35**
8	Right	Audiogram 1	**50**	**50**	**50**	**50**	**70**	15	15	15	20	40
		Audiogram 2	**85**	**85**	**75**	**70**	**70**	20	15	15	15	40
9	Left	Audiogram 1	15	15	10	10	15	**80**	**75**	**60**	**60**	**60**
		Audiogram 2	25	15	10	10	10	**80**	**80**	**70**	**65**	**65**
10	Right	Audiogram 1	**60**	**55**	**35**	**15**	**25**	10	5	10	10	20
		Audiogram 2	**45**	**45**	**40**	**10**	**15**	45	30	10	5	10
11	Right	Audiogram 1	**50**	**55**	**60**	**55**	**50**	15	10	5	5	10
		Audiogram 2	**55**	**65**	**65**	**60**	**60**	20	10	5	5	5

*Note:* The index ear is defined in all cases by the greater average hearing loss compared to the opposite side and PTA threshold of the index ear is marked bold.

In Patients nos. 3 and 6, an increase of ELH in the contralateral ear was also observed. These two patients showed no change in hearing loss in the contralateral ear not only in the investigated frequencies but also in all others. In the cases of progressive hearing loss, a change of the ELH index of at least one point was observed.

**Figure 3:**
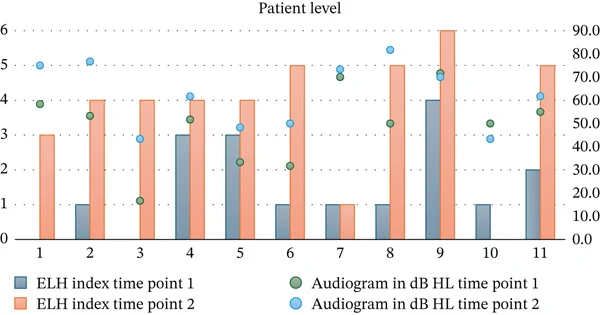
Illustration of ELH index and average hearing loss in form of the audiogram results in dB HL for each patient and both points of time.

In some patients, the grading of ELH and the hearing loss threshold in the index ear were more pronounced in the second attack than during the first. However, both could also show an equal manifestation. In Patients nos. 9 and 10, there was no progression of hearing loss but the ELH changed. These results are shown in Table 4. The correlation between ELH and the average hearing is illustrated for both points of time in Figure 3.

**Table 4:** Correlation between ELH index (without unit) and hearing level in first and second audiogram (both in dB HL).

Patient no	Index Ea	MRI		Audiogram 1		Audiogram 2		Index‐change	Audiogram‐change
		Index 1	Index 2	Right	Left	Right	Left		
				(250 + 500 + 1000 *H* *z*)/3	(250 + 500 + 1000 *H* *z*)/3	(250 + 500 + 1000 *H* *z*)/3	(250 + 500 + 1000 *H* *z*)/3		
1	Right	0	3	58.3	8.3	75.0	11.7	3	16.7
2	Right	1	4	53.3	45.0	76.7	48.3	3	23.4
3	Right	0	4	16.7	16.7	43.3	20.0	4	26.7
4	Left	3	4	13.3	51.7	18.3	61.7	1	10.0
5	Left	3	4	15.0	33.3	15.0	48.3	1	15.0
6	Right	1	5	31.7	15.0	50.0	13.3	4	18.3
7	Left	1	1	18.3	70.0	36.7	73.3	0	3.3
8	Right	1	5	50.0	15.0	81.7	15.0	4	31.7
9	Left	4	6	13.3	71.7	16.7	70.0	2	−1.7
10	Right	1	0	50.0	8.3	43.3	10.0	−1	−6.7
11	Right	2	5	55.0	10.0	61.7	5.0	3	6.7

*Note:* A change in the average hearing loss was defined by ≥ 10 dB HL.

Despite the therapies, changes in ELH index and in the PTA, or both, were observed in all but one of the patients. MD occurred single‐sided in all cases, although the second MRI examination showed an ELH in the ear, which was not the index ear. These patients showed no clinical symptoms in that ear.

All patients underwent two caloric tests. The first caloric test showed an areflexia in relation to the contralateral ear in Patients nos. 3, 8, and 10. In Patient no. 7, there was a bilateral areflexia at the time of first MRI. In earlier examinations shortly after the first symptoms of MD, there was a bilateral response in the caloric testing. All other patients showed a caloric response to stimulation even on the index ear. In 10 out of 11 patients, the caloric tests showed no difference between the first and the second examination.

There was a progressive decrease of caloric response to stimulation in three patients (Patients nos. 3, 4, and 5). Starting with an areflexia on the index ear, Patients nos. 3 and 8 also showed an areflexia in the second test on the contralateral ear. Patient no. 4 showed an areflexia on the index ear in the second testing. In Patient no. 5, there was a regular bilateral stimulation in the first testing. The same patient showed a bilateral areflexia in the second testing. The results are shown in Table 5.

**Table 5:** Correlation between ELH index‐change (without unit), audiogram‐change (in dB HL) and the caloric tests.

Patient no.	Index ear	Index‐change	Audiogram‐change	First caloric test		Second caloric test		Caloric test‐change
				Right	Left	Right	Left	
1	Right	3	16.7	Positive	Positive	Positive	Positive	0
2	Right	3	23.4	Positive	Positive	Positive	Positive	0
3	Right	4	26.7	Negative	Positive	Negative	Negative	0
4	Left	1	10.0	Positive	Positive	Positive	Negative	−1
5	Left	1	15.0	Positive	Positive	Negative	Negative	−1
6	Right	2	18.3	Positive	Positive	Positive	Positive	0
7	Left	0	3.3	Negative	Negative	Negative	Negative	0
8	Right	4	31.7	Negative	Positive	Negative	Negative	0
9	Left	2	−1.7	Positive	Positive	Positive	Positive	0
10	Rechts	−1	−6.7	Negative	Positive	Negative	Positive	0
11	Right	3	6.7	Positive	Positive	Positive	Positive	0

*Note:* There is only a change in the caloric testing in two cases. Under caloric test‐change, 1 represents the change from negative to positive, 0 represents no change, and −1 represents change from positive to negative.

A regression model was applied. The results of average hearing loss in the audiograms were examined for a relationship to the MRI results, the age of the patients, the sex of the patients, and the time intervals between the MRI scans. The application did not reveal any statistically significant relation between hearing loss and age of the patients or sex of the patients or time intervals between MRI scans (see Table 6). However, despite the small sample size, the regression model showed a significant association between the changes in the ELH index on MRI scans and the average hearing loss within the individuals (*p* < 0.001) (see Table 6).

**Table 6:** Linear mixed regression model adjusted for a relationship to the MRI results, the age of the patients, the sex of the patients, and the time intervals between the MRI scans.

Variable	*β*	95%‐confidence interval	*p*
Time of measurement			
*t* = 1	—	—	
*t* = 2	4.0	[−3.7; 12]	0.31
MRI result	5.3	[2.3; 8.2]	< 0.001
Sex			
m	—		
w	−6.4	[−25; 12]	0.49
Age	−0.67	[−1.7; 0.32]	0.18

*Note:* The regression model showed a significance between the ELH and hearing loss.

## 4. Discussion

The visualization of ELH by MRI and its grading has made it possible to investigate the relationship of ELH and the clinical symptoms of MD [3, 14, 17, 18, 21]. Like many patients with MD, our patients also experience fluctuating symptoms, such as variable hearing ability and attacks of vertigo. This is well described in the literature [21, 31, 33].

The same with our cohort, the therapeutic strategies previously used to treat the disease range from lifestyle and dietary changes to invasive surgical procedures. Nearly the whole range described in the literature was used [21, 33–35]. Some of the treatment methods used, such as betahistine, are the subject of controversy in the literature but are still recommended [36–39]. On the other hand, some of these authors observed no influence of the active ingredient dose [38]. In our study, all patients underwent long‐term therapy with betahistine.

A further previous treatment used in all our patients was the systemic application of corticosteroids. In all cases high‐dose methylprednisolone was used. Together with an intratympanic application, it is the next step in a staged therapeutic approach [34]. In our patients, there was no treatment with corticosteroid in‐between the two MRI scans, so there was no influence on the development of the ELH in the MRI scans.

The use of corticosteroids is also controversially discussed in the literature. There are many different treatment protocols [21, 40]. However, there is little evidence to support the benefits of steroids [40]. Kakigi et al. demonstrated that steroids can lead to an increase in ELH [41].

Intratympanic Gentamycin injection offers a further potential therapy. Especially a low‐dose injection is discussed to be another possible treatment. The risk of hearing loss because of the ototoxicity is also regularly discussed [42, 43]. An existing ELH is not expected to affect the effectiveness of the treatment [44]. However, the intratympanic administration of gentamicin may have a reducing effect on ELH [45]. Gentamicin therapy was not used in any of our patients.

Endolymphatic sac surgery is considered to be another treatment option, which was performed in one of our patients before the first MRI scan. Distinctions can be made between decompression and drainage or blockage surgery. All these are discussed as possible therapy options in MD [8, 20, 33, 35, 46]. He et al. showed that endolymphatic duct blockage has an impact on the ELH grading in MRI [33]. In the one described case in our study, the ELH increased over time even after a performed surgery.

Another described surgical intervention is the vestibular neurectomy. With this treatment, a reduction in hydrops was observed [47]. This treatment was not performed on our patients.

Previous research has shown that in MD, a higher grade of ELH on MRI imaging is also associated with more pronounced symptoms or vestibulocochlear dysfunction [12, 21, 48–50].

Despite the small number of patients, there was a significance between the increase of ELH and symptoms corresponding to MD.

In our cohort, there were nine increasing ELH, one stable ELH, and one decreasing ELH. Among the nine patients with an increasing ELH, there were seven patients with an also increasing average hearing loss and two with a stable average hearing loss.

In the patient with the stable ELH, the average hearing loss was stable, too. The patient with the decreased ELH showed a decreasing hearing loss, too. All in all, 9 out of 11 patients showed an average hearing loss which followed the ELH.

In 9 out of 11 patients, the findings of the caloric test did not change between the first and the second testing. In two cases, the caloric test changed from positive to negative. In both cases, the ELH and the average hearing loss increased. There is a controversial discussion about this observation in the literature. In some studies, a correlation between the grading of ELH and the results of caloric examination was found [51]. Other studies denied these findings [48].

To objectively assess the occurrence of dizziness, a dizziness diary can be used. Here, a distinction can be made between an “all‐or‐nothing” principle: dizziness either occurs or it does not. Some authors have devised a 5‐point scale to assess the severity of subjective dizziness [52]. However, changes in the vertigo score do not correlate with ELH [53]. However, no such scale was used in our patients. The next episode marked the time of the second hospitalization.

Our study also supports other observations showing that ELH can also occur on the contralateral side of the index ear with increasing disease duration [20, 21, 34]. It is possible that Ménière‐like symptoms can occur with these ears in the future. There was no change in hearing loss at all frequencies in the two patients with an increase of ELH in the contralateral ear.

Limitations of the study are the lack of predictability of a new attack and the small number of patients. Especially the small sample size can lead to uncertainties in the overall statement. It is possible that correlations between the parameters examined were not discovered, even though they might exist. In our study no relationship between the hearing loss and age or gender of the patients or time intervals between the MRI scans was found. Therefore, Type II errors are potentially possible.

The second MRI could not be performed as planned and should be performed within a short time after the onset of the seizure if possible. The data furthermore showed a significance between ELH and the average hearing loss. Perhaps a higher number of patients could also lead to a more pronounced result.

In this study, vestibular function was investigated using the caloric test. For further investigations of the relationship between ELH and vestibular function, the video head impulse test (vHIT) and vestibular‐evoked myogenic potentials (VEMPs) should also be used.

All in all, there was a longitudinal observation of patients suffering from MD using a repeated MRI‐based ELH grading as well as the parallel collected clinical data.

## 5. Conclusion

Our study shows that MRI‐based ELH, like MD symptoms, is not static. The results suggest that MD symptoms change in many cases similar to the MRI pattern of ELH. This semble to be independent of whether the ELH is in an increased or unchanged state. However, the available data are based entirely on observations, as there is considerable variability among patients. For this reason, no causal relationships or conclusions regarding the efficacy of a treatment can be drawn.

## Author Contributions

C.J.P.: writing, idea, correction, and final approval. H‐B.G.: analysis, correction, and final approval. A.K.: analysis, correction, and final approval. C.R.: analysis, correction, and final approval. L‐U.S.: analysis, correction, and final approval. D.V.: analysis, correction, and final approval. I.T.: idea, analysis, correction, and final approval.

## Funding

No funding was received for this manuscript. Open Access funding enabled and organized by Projekt DEAL.

## Ethics Statement

The study was approved by the Ethical Committee of the Ärztekammer Westfalen‐Lippe, University of Münster Faculty of Medicine, Bielefeld University, Medical Faculty (Reference: 2022‐314‐f‐S).

## Conflicts of Interest

The authors declare no conflicts of interest.

## Supporting information


**Supporting Information** Additional supporting information can be found online in the Supporting Information section. The supporting information contains the blinded original data per each included patient.

## Data Availability

The data used to support this study′s findings are available from the corresponding author upon request.
