# Supplementary material for: Observation of MRI‐Based Endolymphatic Hydrops and Clinical Symptoms in Patients With Definite Meniere′s Disease Over Time: A Case Series
Source: Biomed Res Int. 2026 Sep 3;2026:9060023. doi: 10.1155/bmri/9060023 (PMC13542573; doi:10.1155/bmri/9060023)
Supplement: Supplementary file 1 [file BMRI-2026-9060023-s001.pdf]

| | | | | | | | | | | | ear right | | ear |
|---|---|---|---|---|---|---|---|---|---|---|---|---|---|
| Nr | Pat.-ID | Sex | Age at | Index ear | duration in d | Corticosteroid infusion | Tinnitus | Betahistin | Saccotomie | MRI 1 | vestibular | cochlear | vestibular |
| 1 | 2232641 | m | 52,0 | right | 755 | yes | yes | yes | yes | 11.07.2018 | **0** | **0** | 0 |
| 2 | 2085864 | m | 65,8 | right | 2226 | yes | yes | yes | no | 16.09.2016 | **1** | **0** | 0 |
| 3 | 2128544 | m | 54,5 | right | 2053 | yes | yes | yes | yes | 04.11.2015 | **0** | **0** | 0 |
| 4 | 557040 | m | 60,1 | left | 392 | yes | yes | yes | yes | 05.06.2020 | 0 | 0 | **2** |
| 5 | 446456 | f | 58,1 | left | 701 | yes | yes | yes | no | 20.01.2020 | 0 | 0 | **2** |
| 6 | 2324368 | f | 50,4 | right | 553 | yes | yes | yes | no | 02.07.2021 | **1** | **0** | 0 |
| 7 | 2267268 | f | 35,4 | left | 413 | yes | yes | yes | no | 04.02.2021 | 0 | 0 | **0** |
| 8 | 2337501 | m | 42,5 | right | 351 | yes | yes | yes | no | 14.12.2021 | **0** | **1** | 0 |
| 9 | 2295669 | m | 48,7 | left | 399 | yes | yes | yes | no | 19.06.2020 | 0 | 0 | **2** |
| 10 | 506802 | f | 50,3 | right | 1108 | yes | yes | yes | no | 04.08.2021 | **1** | **0** | 1 |
| 11 | 2331500 | m | 55,1 | right | 570 | yes | yes | yes | no | 08.11.2021 | **1** | **1** | 0 |

| left | | | ear right | | ear left | | | Index- | | | | right | |
|---|---|---|---|---|---|---|---|---|---|---|---|---|---|---|
| cochlear | Index | MRI 2 | vestibular | cochlear | vestibular | cochlear | Index | Change | 0,25 kHz | 0,5 kHz | 1 kHz | 2 kHz | 4 kHz |
| 0 | 0 | 04.08.2020 | 1 | 2 | 0 | 0 | 3 | 3 | 60 | 55 | 60 | 40 | 35 |
| 0 | 1 | 21.10.2022 | 2 | 2 | 0 | 0 | 4 | 3 | 55 | 55 | 50 | 30 | 35 |
| 0 | 0 | 18.06.2021 | 2 | 2 | 0 | 1 | 4 | 4 | 20 | 15 | 15 | 20 | 40 |
| 1 | 3 | 02.07.2021 | 0 | 0 | 2 | 2 | 4 | 1 | 15 | 10 | 15 | 10 | 10 |
| 1 | 3 | 21.12.2021 | 0 | 0 | 2 | 2 | 4 | 1 | 15 | 15 | 15 | 20 | 25 |
| 0 | 1 | 06.01.2023 | 1 | 2 | 0 | 2 | 3 | 2 | 40 | 30 | 25 | 25 | 30 |
| 1 | 1 | 24.03.2022 | 0 | 0 | 0 | 1 | 1 | 0 | 10 | 20 | 25 | 15 | 10 |
| 0 | 1 | 30.11.2022 | 3 | 2 | 0 | 0 | 5 | 4 | 50 | 50 | 50 | 50 | 70 |
| 2 | 4 | 23.07.2021 | 0 | 0 | 3 | 3 | 6 | 2 | 15 | 15 | 10 | 10 | 15 |
| 0 | 1 | 16.08.2024 | 0 | 0 | 1 | 0 | 0 | -1 | 60 | 55 | 35 | 15 | 25 |
| 0 | 2 | 01.06.2023 | 3 | 2 | 0 | 0 | 5 | 3 | 50 | 55 | 60 | 55 | 50 |

| **Audiogram 1** | | | | | | | | | | | | **Audiogram 2** | | |
|---|---|---|---|---|---|---|---|---|---|---|---|---|---|---|
| | | | | **left** | | | | | | **right** | | | | |
| **(250+500+1000 Hz)/3** | **0,25 kHz** | **0,5 kHz** | **1 kHz** | **2 kHz** | **4 kHz** | **(250+500+1000 Hz)/3** | **0,25 kHz** | **0,5 kHz** | **1 kHz** | **2 kHz** | **4 kHz** | **(250+500+1000 Hz)/3** | **0,25 kHz** | **0,5 kHz** |
| **58,3** | 10 | 5 | 10 | 10 | 20 | 8,3 | **80** | **75** | **70** | **55** | **65** | **75,0** | 10 | 10 |
| **53,3** | 40 | 50 | 45 | 35 | 35 | 45,0 | **85** | **75** | **70** | **65** | **75** | **76,7** | 45 | 50 |
| **16,7** | 20 | 15 | 15 | 20 | 40 | 16,7 | **50** | **45** | **35** | **45** | **55** | **43,3** | 20 | 20 |
| 13,3 | **50** | **50** | **55** | **45** | **55** | **51,7** | 25 | 15 | 15 | 15 | 20 | 18,3 | **60** | **60** |
| 15,0 | **40** | **30** | **30** | **35** | **35** | **33,3** | 15 | 15 | 15 | 20 | 30 | 15,0 | **55** | **50** |
| **31,7** | 15 | 15 | 15 | 20 | 25 | 15,0 | **50** | **50** | **50** | **45** | **50** | **50,0** | 10 | 15 |
| 18,3 | **70** | **70** | **70** | **50** | **40** | **70,0** | 40 | 40 | 30 | 20 | 15 | 36,7 | **75** | **75** |
| **50,0** | 15 | 15 | 15 | 20 | 40 | 15,0 | **85** | **85** | **75** | **70** | **70** | **81,7** | 20 | 15 |
| 13,3 | **80** | **75** | **60** | **60** | **60** | **71,7** | 25 | 15 | 10 | 10 | 10 | 16,7 | **80** | **80** |
| **50,0** | 10 | 5 | 10 | 10 | 20 | 8,3 | **45** | **45** | **40** | **10** | **15** | **43,3** | 45 | 30 |
| **55,0** | 15 | 10 | 5 | 5 | 10 | 10,0 | **55** | **65** | **65** | **60** | **60** | **61,7** | 20 | 10 |

| left | | | | Audiogram- | First caloric test | | Second caloric test | | caloric test- |
|---|---|---|---|---|---|---|---|---|---|
| **1 kHz** | **2 kHz** | **4 kHz** | **(250+500+1000 Hz)/3** | **Tendency** | **right** | **left** | **right** | **left** | **Change** |
| 15 | 10 | 30 | 11,7 | **16,7** | **positive** | positive | positive | positive | **0** |
| 50 | 55 | 55 | 48,3 | **23,4** | **positive** | positive | positive | positive | **0** |
| 20 | 30 | 45 | 20,0 | **26,7** | **negative** | positive | negative | negative | **0** |
| **65** | **65** | **65** | **61,7** | **10,0** | positive | **positive** | positive | **negative** | **-1** |
| **40** | **40** | **85** | **48,3** | **15,0** | positive | **positive** | negative | **negative** | **-1** |
| 15 | 15 | 15 | 13,3 | **18,3** | **positive** | positive | **positive** | positive | **0** |
| **70** | **60** | **35** | **73,3** | **3,3** | negative | **negative** | negative | **negative** | **0** |
| 15 | 15 | 40 | 15,0 | **31,7** | **negative** | positive | **negative** | negative | **0** |
| **70** | **65** | **65** | **70,0** | **-1,7** | positive | **positive** | positive | **positive** | **0** |
| 10 | 5 | 10 | 10,0 | **-6,7** | **negative** | positive | **negative** | positive | **0** |
| 5 | 5 | 4 | 5,0 | **6,7** | positive | positive | positive | positive | **0** |
